# High prevalence of intestinal colonization by beta-lactam resistant Enterobacterales in patients admitted to an emergency department in Lima, Peru

**DOI:** 10.1016/j.nmni.2026.101824

**Published:** 2026-08-04

**Authors:** Gustavo Gutierrez Batallanos, Alvaro Madrid, Edwin Varillas, Noemi Hinostroza, Fiorella Krapp

**Affiliations:** a School of Medicine, Universidad Peruana Cayetano Heredia, 430 Honorio Delgado Avenue, Lima, 15102, Peru; b Instituto de Medicina Tropical Alexander von Humboldt, Universidad Peruana Cayetano Heredia, 430 Honorio Delgado Avenue, Lima, 15102, Peru

**Keywords:** Carbapenem-resistant enterobacterales, Beta-lactam resistance, Multidrug resistance, Bacterial, Colonization, Peru

## Abstract

**Objectives:**

To determine the prevalence of intestinal colonization by extended-spectrum beta-lactamase-producing Enterobacterales (ESBL-E) and carbapenem-resistant Enterobacterales (CRE) among patients admitted to the emergency department of a tertiary care hospital in Lima, Peru. Factors associated with colonization were also evaluated.

**Methods:**

A cross-sectional study was conducted from December 2019 to March 2020. Patients were enrolled on one to two randomly selected days per week. A rectal or fecal swab was collected, and a standardized questionnaire was administered. Samples were cultured on chromogenic media (ChromAgar ESBL and ChromAgar mSuperCarba) and disc diffusion methods were used for confirmation of ESBL production and carbapenem resistance. The association of epidemiological and clinical factors with ESBL-E and CRE colonization was analyzed using bivariate generalized linear models with a Poisson distribution and robust variance.

**Results:**

A total of 148 patients were sampled. The prevalence of ESBL-E and CRE colonization was 73.6% (109/148) and 8.1% (12/148), respectively. Hospitalization during the previous year (PR = 1.23, 95% CI: 1.02-1.49) was associated with ESBL-E colonization.

**Conclusions:**

A high prevalence of ESBL-E intestinal colonization was found among patients with and without prior healthcare exposure admitted to the emergency department of a tertiary care hospital in Lima.

## Highlights

• Overall high prevalence of ESBL-E rectal colonization in an emergency department in Lima.
• In the proxy group for community acquired ESBL-E colonization the prevalence was markedly high (80%).
• CRE colonization was present in 8.1% of emergency department patients, supporting the need for enhanced infection prevention measures at hospital admission.
• The absence of CRE in the community proxy group suggests that CRE colonization remains largely healthcare-associated.

## 1 Introduction

In recent years, the World Health Organization (WHO) included carbapenem-resistant Enterobacterales (CRE) and extended-spectrum beta-lactamase producing Enterobacterales (ESBL-E) in the list of critical priority pathogens that require continuous surveillance and newer treatment options, as they pose a serious threat to global health [1].

CRE and ESBL-E infections are associated with higher mortality rates and hospital costs than infections caused by non-resistant bacteria [2,3]. Approximately 16.5% of hospitalized patients colonized with CRE developed an infection, most frequently in the lungs and urinary tract [4]. In contrast, among patients colonized with ESBL-E, the reported progression to infection varies considerably according to the population and the duration of follow-up, ranging from 1.4% during hospitalization in a general ward to 21.5% in a population-wide cohort (including non-hospitalized and hospitalized patients) with long-term follow-up [5,6]. Furthermore, CRE or ESBL-E colonization in asymptomatic patients can become a source of further spread within and outside the hospital [4,7]. Notably, persistent colonization can be detected in up to a third of patients even after one year of follow-up [8].

According to a 2019 global estimate of antimicrobial resistance burden, Latin America is one of the regions with the highest rates of ESBL-E worldwide [3]. Within the region, Peru exhibits the highest prevalence of ESBL-E, as 40-50% of *Escherichia coli* isolates and 60-70% *Klebsiella pneumoniae* isolates are resistant to third generation cephalosporins [3]. In contrast, the CRE prevalence in Peru was estimated to be <5%, lower than many other countries in Latin America [3]. These estimates reflect the frequency of resistance among isolates recovered from infected patients. However, the frequency of intestinal colonization by these beta-lactam resistant Enterobacterales is less well known [9,10]. Since colonization typically precedes infection [ 4], understanding the true extent of colonization by both CRE and ESBL-E is critical. Therfore, this study aimed to determine the prevalence of ESBL-E and/or CRE intestinal colonization in patients admitted to the emergency department of a tertiary care hospital in Lima and to explore the demographic and clinical factors associated with colonization.

## 2 Methods

This cross-sectional study was performed in the emergency department of Hospital Cayetano Heredia, a 375-bed public tertiary care hospital, located in Lima, Peru. The emergency department has 60 beds; thus, patients may remain for several days while waiting for an available bed in the inpatient wards.

Patient enrollment was conducted on one to two randomly selected days per week from December 2019 to March 2020. All adult patients admitted to the ED who agreed to participate in the study were included. Pregnant patients, and patients who did not have a fecal sample available or had contraindications for rectal swab, such as severe neutropenia, proctitis, lower gastrointestinal bleeding or severe coagulation disorder, were excluded. The estimated sample sizes, with a precision of 5%, were 73 and 288 for an expected CRE prevalence of 5% [3,11] and an ESBL-E prevalence of 25% [12], respectively. Enrollment was terminated early due to the restrictions implemented during the COVID-19 pandemic. Demographic and epidemiological data were collected using a questionnaire and through review of medical records. Collected data included sex, age, time since admission, comorbidities, previous healthcare exposure (including hospitalization, surgery or dialysis within the previous year), previous outpatient visits, international travel and contact with a previously hospitalized relative within the previous year, and antibiotic treatments in the preceding 6 months before the collection of the sample.

Each patient provided a single fecal sample which was collected either via rectal or fecal swabs. Samples were transported in Cary-Blair medium. Each sample was inoculated onto two chromogenic selective media: ChromAgar ESBL (CHROMagarTM) and ChromAgar mSuperCARBA (CHROMagarTM) and incubated aerobically at 37°C four 24 h. From each positive plate, one representative colony of each morphotype compatible with Enterobacterales was selected and subcultured onto Trypticase Soy Agar for further analysis. Colonies with chromogenic appearances compatible with *E. coli* or *K. pneumoniae* were classified as presumptive species, while atypical isolates were evaluated using conventional biochemical test (triple sugar iron [TSI], citrate, lysine iron agar [LIA], and motility-indole-ornithine [MIO] [13]. Isolates recovered from CHROMagar ESBL were tested by the combined disc diffusion method to confirm ESBL production, whereas isolates recovered from CHORMagar mSuperCARBA were tested by disk diffusion using imipenem, meropenem and ertapenem to confirm carbapenem resistance according to the Clinical and Laboratory Standards Institute (CLSI) guidelines [14]. The prevalence of ESBL-E and CRE colonization was determined as the number of patients with at least one confirmed ESBL-E or CRE isolate divided by the total number of participants. To account for potential timing of acquisition, prevalence was stratified by time since hospital admission: ≤ 48 h of admission (more likely reflecting acquisition prior to admission) and > 48 h (which may include both acquisition prior to admission or early during ED hospitalization). In addition, a subgroup analysis was performed among patients admitted for ≤48 h with no prior healthcare exposure, serving as a proxy for community acquisition. Categorical variables were presented as frequencies (percentages), and numerical variables with non-normal distribution as median and interquartile ranges (IQR). All variables were compared between ESBL-E colonized and non-colonized patients using Fisher's exact or Mann-Whitney U tests, as appropriate.

Exploratory regression analyses were conducted using Generalized Linear Model (GLM) with a Poisson distribution, a log link function, and robust standard error to calculate prevalence ratios (PR). Ninety-five percent confidence intervals (95%CIs) were reported, and p-values < 0.05 were considered statistically significant.

To address missing data in the variable selection process, single imputation was applied by replacing missing 'Unknown' values with the modal category 'No'. As a first step, bivariate analyses were performed using the complete case dataset (n = 130), and all variables with a p-value < 0.1 were considered for manual forward selection to develop a multivariable model. Model comparisons were assessed using likelihood-ratio (LR) tests with p < 0.05 as the criterion for variable inclusion. To evaluate the robustness of variable selection, the same forward selection procedure was applied to the imputed dataset (n = 148). Final model estimates were reported from the imputed dataset because it provided greater precision. Statistical analysis was performed using Stata® software, v17 (StataCorp 17.1, College Station, Texas, USA). This study was approved by the ethical committee of Hospital Cayetano Heredia and Universidad Peruana Cayetano Heredia.

## 3 Results

**Fig. 1:**
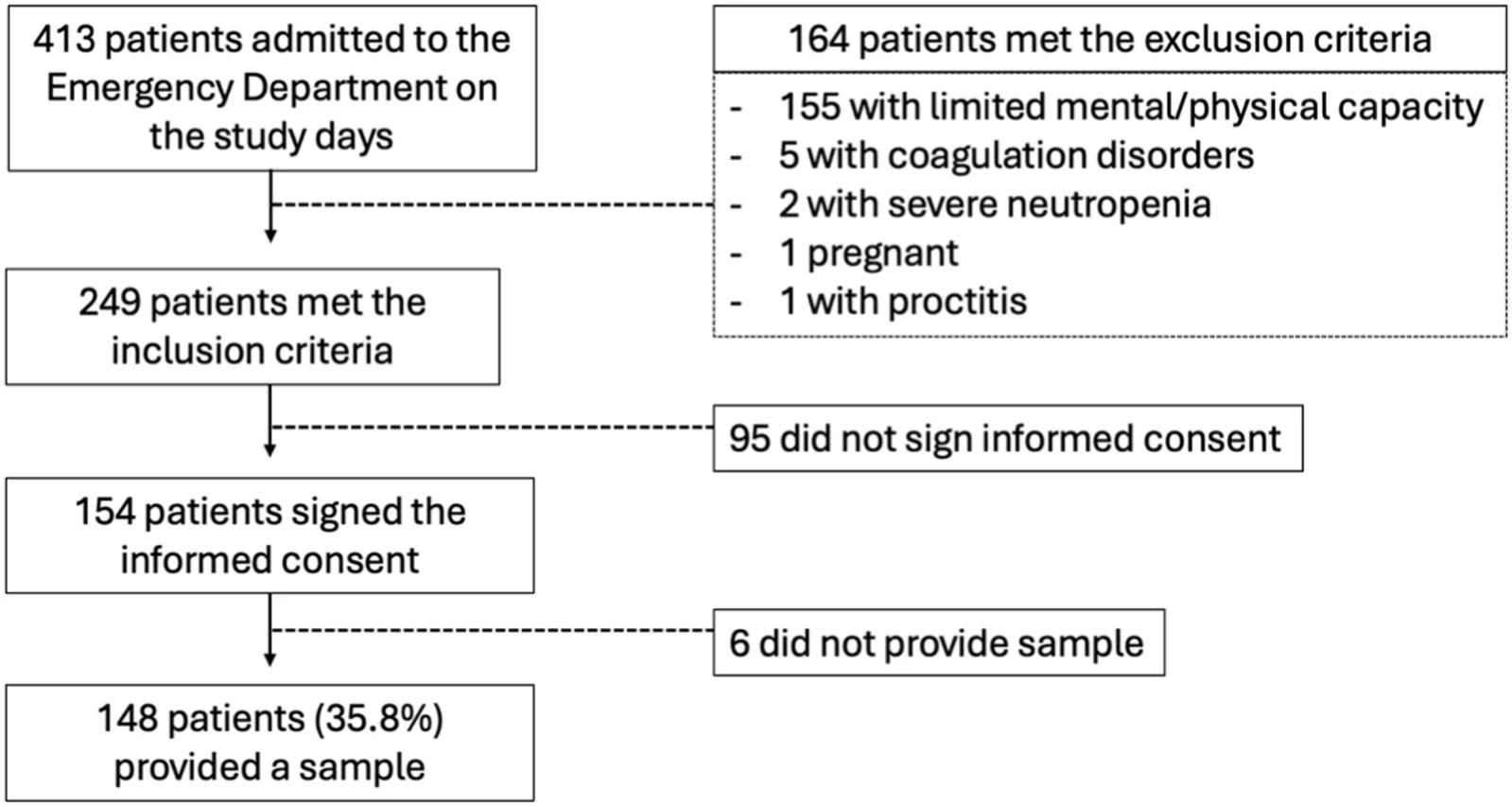
Patient enrollment and exclusion process for sample collection in the Emergency Department of a tertiary care hospital in Lima, Peru, from December 2019 to March 2020.

During the fifteen enrollment days within the study period, a total of 413 patients were admitted to the emergency department (ED). Of these, 249 (60.3%) met the inclusion criteria, 154 (37.3%) agreed to participate in the study, and 148 provided a fecal or rectal sample, representing 35.8% of all admitted patients. The main reason for exclusion was inability to provide informed consent due to impaired consciousness/cognitive status (155 out of 166 excluded patients) (Fig. 1). The median time between ED admission and enrollment was 3.4 days (IQR 2-7.8), and 37 (25%) were enrolled within the first 48 h of admission.

Among 148 patients, the median age was 64 years (IQR 53-76.5), and 48% (n = 71) were female. Overall, 66.9% (n = 99) had at least one comorbidity, and 39.9% (n = 59) reported using antibiotics within the previous month. Furthermore, 41.9% (n = 62) reported previous healthcare exposure, including 19.6% (n = 29) who had been hospitalized in the previous 3 months (Table 1).

**Table 1:** Characteristics of patients with ESBL-E and CRE colonization in the Emergency Department of a tertiary care hospital in Lima, Peru, 2019-2020.

Variable	Total n = 148 (%)	ESBL-E colonization n = 109 (%)	CRE colonization n = 12 (%)	No colonization n = 36 (%)
Age,median (IQR)	64 (53-76.5)	65 (54-81)	73.5 (63.5-82)	61 (47-69)
≤ 75	108 (73.0)	76 (69.7)	6 (50.0)	30 (83.3)
> 75	40 (27.0)	33 (30.3)	6 (50.0)	6 (16.7)
Time following admission (hours) (IQR)	82 (48-188)	75 (46-188)	184.5 (50-279.5)	86.5 (50.5-180)
≤48 h	37 (25.0)	30 (27.5)	3 (25.0)	7 (19.4)
> 48 h	111 (75.0)	79 (72.5)	9 (75.0)	29 (80.6)
Sex				
Male	77 (52.0)	54 (49.5)	3 (25.0)	23 (63.9)
Female	71 (48.0)	55 (50.5)	9 (75.0)	13 (36.1)
At least 1 comorbidity				
Yes	99 (66.9)	73 (67.0)	8 (66.7)	23 (63.9)
No	49 (33.1)	36 (33.0)	4 (33.3)	13 (36.1)
Comorbidities				
Diabetes	46 (31.1)	33 (30.3)	4 (33.3)	11 (30.6)
Recurrent UTI	8 (5.4)	7 (6.4)	0 (0.0)	1 (2.8)
CLD	14 (9.5)	13 (11.9)	0 (0.0)	1 (2.8)
CPD	17 (11.5)	15 (13.8)	2 (16.7)	2 (5.6)
CKD	34 (23.0)	23 (21.1)	2 (16.7)	10 (27.8)
Immunosuppression	3 (2.0)	2 (1.8)	0 (0.0)	1 (2.8)
HIV	3 (2.0)	2 (1.8)	0 (0.0)	1 (2.8)
Cancer	16 (10.8)	12 (11.0)	2 (16.7)	3 (8.3)
Previous healthcare exposure				
Yes	62 (41.9)	48 (44)	5 (41.7)	13 (36.1)
No	76 (51.3)	54 (49.5)	6 (50.0)	20 (55.6)
Unknown	10 (6.7)	7 (6.4)	1 (8.3)	3 (8.3)
Hospitalization within previous year				
Yes	50 (33.8)	42 (38.5)	5 (41.7)	7 (19.4)
No	88 (59.5)	60 (55.1)	6 (50.0)	26 (72.2)
Unknown	10 (6.8)	7 (6.4)	1 (8.3)	3 (8.3)
Dialysis				
Yes	22 (14.9)	15 (13.8)	1 (8.3)	7 (19.4)
No	116 (78.4)	87 (79.8)	10 (83.3)	26 (72.2)
Unknown	10 (6.8)	7 (6.4)	1 (8.3)	3 (8.3)
Previous surgery				
Yes	8 (5.4)	5 (4.6)	0 (0.0)	3 (8.3)
No	130 (87.8)	97 (89.0)	11 (91.7)	30 (83.3)
Unknown	10 (6.8)	7 (6.4)	1 (8.3)	3 (8.3)
Previous outpatient visit				
Yes	52 (35.1)	43 (39.5)	4 (33.3)	9 (25.0)
No	78 (52.7)	53 (48.6)	5 (41.7)	23 (63.9)
Unknown	18 (12.2)	13 (11.9)	3 (25.0)	4 (11.1)
Last time of hospitalization				
Within 1 month	17 (34.0)	14 (33.3)	2 (40.0)	3 (42.9)
Within 2-3 months	12 (24.0)	9 (21.4)	1 (20.0)	2 (28.6)
Within 4-5 months	9 (18.0)	8 (19.1)	1 (20.0)	1 (14.3)
More than 6 months	11 (22.0)	10 (23.8)	0 (0.0)	1 (14.3)
Unknown	1 (2.0)	1 (2.4)	1 (20.0)	0 (0.0)
Antibiotics within previous month				
Yes	59 (39.9)	43 (39.5)	5 (41.7)	15 (41.7)
No	71 (48.0)	53 (48.6)	6 (50.0)	16 (44.4)
Unknown	18 (12.2)	13 (11.9)	1 (8.3)	5 (13.9)
Antibiotics within 2- 6 previous months				
Yes	39 (26.4)	30 (27.5)	2 (16.7)	9 (25.0)
No	84 (56.8)	59 (54.1)	8 (66.7)	23 (63.9)
Unknown	25 (16.9)	20 (18.4)	2 (16.7)	4 (11.1)

UTI, Urinary Tract Infection; CLD, Chronic Liver Disease; CPD, Chronic Pulmonary Disease; CKD, Chronic Kidney Disease; HIV, Human immunodeficiency virus; IQR, Interquartile Range; CRE, Carbapenem-resistant Enterobacterales; ESBL-E, ESBL-producing Enterobacterales. There were 9 co-colonized with ESBLE and CRE individuals in the study. Previous healthcare exposure: includes either hospitalization, surgery or dialysis within the previous year. Previous outpatient visits within the previous year were assessed separately and not considered a healthcare exposure.

### 3.1 ESBL-E and CRE colonization

Following incubation on the ESBL screening medium, 121 patient samples showed positive growth; on the CRE screening medium, 47 showed positive growth. From these samples a total of 128 isolates were confirmed as ESBL-E and 16 as CRE.

The overall prevalence of ESBL-E and CRE colonization was 73.6% (109/148, 95 CI: 65.8 – 80.5%) and 8.1% (12/148, 95 CI: 0.04-13.7%) respectively. In the proxy group for community-acquired colonization, 0% (0/20) and 80% (16/20) had CRE and ESBLE-E colonization, respectively (Supplementary Table 1). Eighteen ESBL-E colonized patients had more than one bacterial species identified; whereas four CRE colonized patients had more than one species identified (Supplementary Table 2), resulting in a total of 128 ESBL-E and 16 CRE bacterial isolates recovered. Presumptive *E. coli* was the predominant ESBL-E isolate (105/128) followed by presumptive *K. pneumoniae* (23/128). In contrast, presumptive *K. pneumoniae* was the predominant CRE isolate (9/16) followed by presumptive *E. coli* (6/16), and *Enterobacter spp*. (1/16).

**Fig. 2:**
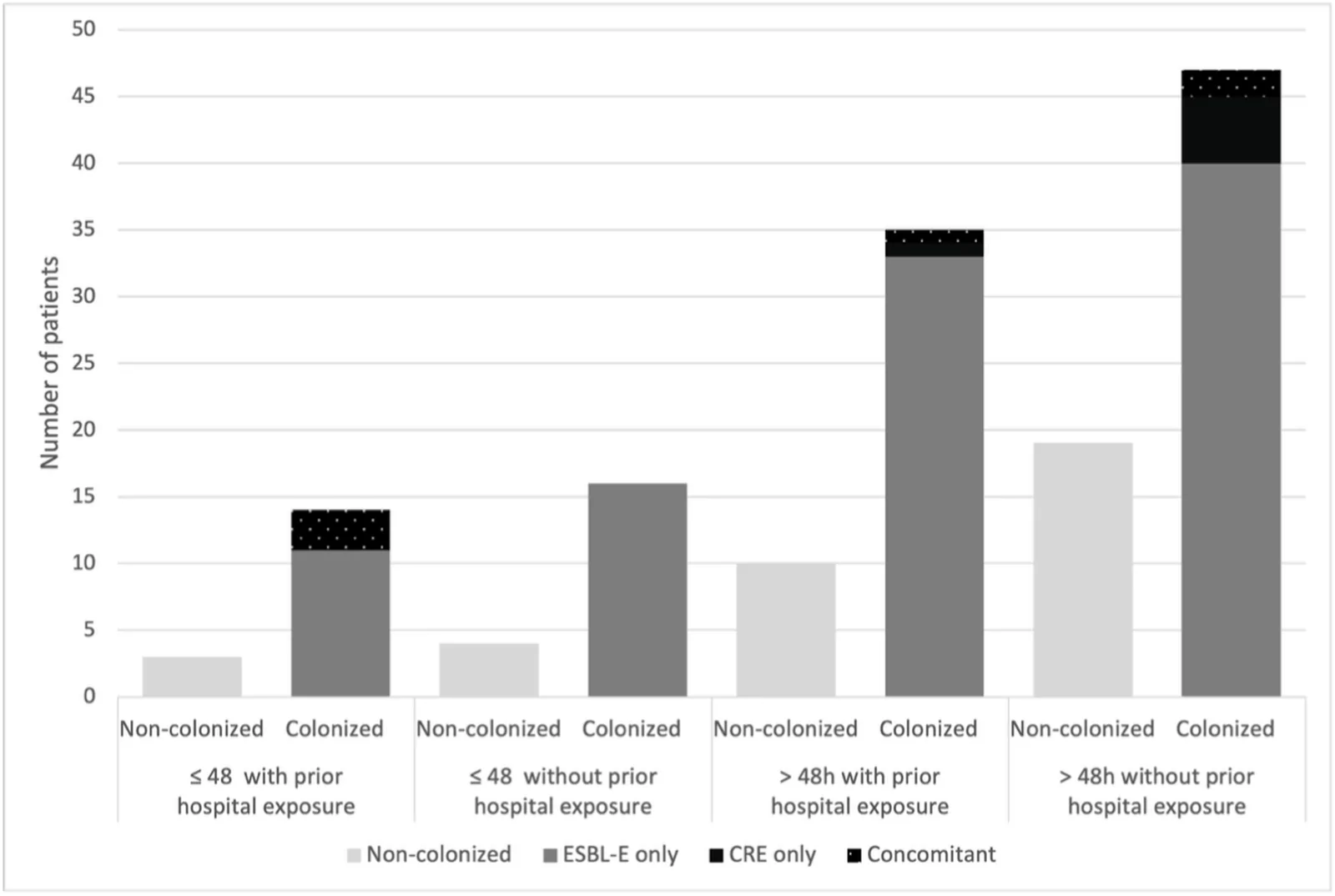
ESBL-E and CRE colonization based on time since emergency department admission and prior healthcare exposure.

The prevalence of ESBL-E and CRE colonization in patients with ≤ 48 h of ED admission was 81.1% (30/37) and 8.1% (3/37), respectively (Fig. 2). Among ESBL-E colonized patients within ≤48 h, 53.3% (16/30) reported no prior healthcare exposure, 56.3% (9/16) of these reported use of antibiotics such as cephalosporins, quinolones, sulfonamides or penicillin within the previous 6 months before the sampling. In contrast, all three CRE-colonized enrolled within ≤48 h had simultaneous ESBL-E colonization and all reported hospitalization during the previous month and previous use of parenteral antibiotics (e.g., vancomycin, cephalosporins).

**Supplementary Fig. 1:**
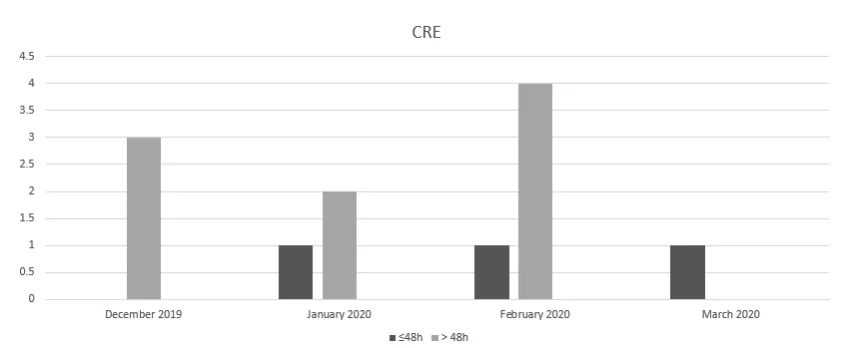
CRE colonization based on time since emergency department admission CRE: Carbapenem-resistant Enterobacterales. ≤48 h: Number of patients colonized within the first 48 h >48 h: Number of patients colonized with more than 48 h.

**Supplementary Fig. 2:**
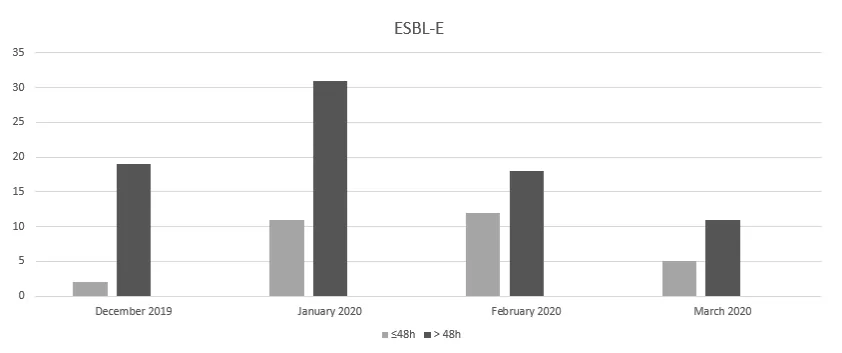
ESBL-E colonization based on time since emergency department admission ESBL-E: ESBL-producing Enterobacterales. ≤48 h: Number of patients colonized within the first 48 h >48 h: Number of patients colonized with more than 48 h.

The frequency of ESBL-E and CRE colonization in patients with > 48 h of ED admission was 71.7% (79/111) and 8.1% (9/111), respectively. Six out of nine CRE-colonized patients had simultaneous ESBL-E colonization. Only three out of nine CRE-colonized patients reported previous healthcare exposure or antibiotic use in the previous month. Whereas, among ESBL-E colonized patients, 43% (34/79) reported previous healthcare exposure, and 34.1% (27/79) reported antibiotic use in the previous month (Fig. 2, Supplementary Figs. 1 and 2).

### 3.2 Factors associated with ESBL-E colonization

In the bivariate analysis (Table 2), hospitalization within the previous year, chronic liver disease, and chronic pulmonary disease were significantly associated with ESBL-E colonization (p < 0.05), while age group (≤75 vs. > 75 years) and previous outpatient visits also met the prespecified criterion (p < 0.1) for consideration in the multivariable analysis. Only prior hospitalization within the previous year was retained in the final model (Supplementary Table 3). Individuals hospitalized within the past year had 23% higher prevalence of ESBL-E colonization (PR = 1.23, 95% CI: 1.02–1.49, p = 0.026).

**Table 2:** Bivariate analysis of associated factors with ESBL-E colonization in patients admitted to the emergency department of a tertiary care hospital in Lima, Peru, 2019-2020.

Variable	ESBL-E colonized N = 109, n (%)	Non ESBL-E colonized N = 39, n (%)	cPR (95% CI)	p- value^a^
Age (years)				
≤ 75	76 (69.7)	32 (82.1)	Ref	
> 75	33 (30.3)	7 (17.9)	1.17 (0.97-1.42)	0.098
Time following admission				
≤48 h	30 (27.5)	7 (18.0)	Ref	
> 48 h	79 (72.5)	32 (82.0)	0.88 (0.72-1.07)	0.193
Sex				
Male	54 (49.5)	23 (59.0)	0.90 (0.75-1.10)	0.312
Female	55 (50.5)	16 (41.0)	Ref	
At least one comorbidity				
Yes	73 (67.0)	26 (66.7)	1.00 (0.82-1.23)	0.972
No	36 (33.0)	13 (33.3)	Ref	
Comorbidities				
Diabetes	33 (30.3)	13 (33.3)	0.96 (0.78-1.19)	0.729
Recurrent UTI	7 (6.4)	1 (2.6)	1.20 (0.91-1.59)	0.203
CLD	13 (11.9)	1 (2.6)	1.30 (1.08-1.55)	0.005
CPD	15 (13.8)	2 (5.1)	1.23 (1.00-1.51)	0.048
CKD	23 (21.1)	11 (28.2)	0.90 (0.69-1.16)	0.404
Immunosuppression	2 (1.8)	1 (2.6)	0.90 (0.40-2.03)	0.806
HIV	2 (1.8)	1 (2.6)	0.90 (0.69-1.16)	0.806
Cancer	12 (11.0)	4 (10.3)	1.02 (0.75-1.38)	0.895
Previous outpatient visit				
Yes	43 (44.8)	9 (26.5)	1.22 (1.00-1.48)	0.051
No	53 (55.2)	25 (73.5)	Ref	
Previous healthcare exposure				
Yes	48 (47.1)	14 (38.9)	1.09 (0.89-1.33)	0.394
No	54 (52.9)	22 (61.1)	Ref	
Dialysis				
Yes	15 (14.7)	7 (19.4)	0.91 (0.97-1.23)	0.541
No	87 (85.3)	29 (80.6)	Ref	
Previous surgery*				
Yes	5 (4.9)	3 (8.3)	0.84 (0.48-1.45)	0.526
No	97 (95.1)	33 (91.7)	Ref	
Hospitalization within previous year				
Yes	42 (41.2)	8 (22.2)	1.23 (1.02-1.49)	0.029
No	60 (58.8)	28 (77.8)	Ref	
Antibiotics within previous month				
Yes	43 (44.8)	16 (47.1)	0.98 (0.79-1.20)	0.821
No	53 (55.2)	18 (52.9)	Ref	
Antibiotics within previous 6 months				
Yes	30 (33.7)	9 (26.5)	1.10 (0.88-1.37)	0.422
No	59 (66.3)	25 (73.5)	Ref	

cPR, crude prevalence ratio; 95%CI, 95% confidence interval; UTI, Urinary Tract Infection; CLD, Chronic Liver Disease; CPD, Chronic Pulmonary Disease; CKD, Chronic Kidney Disease; CRE, Carbapenem-resistant Enterobacterales; ESBL-E, ESBL-producing Enterobacterales. Previous healthcare exposure: includes either hospitalization, surgery or dialysis within the previous year. Previous outpatient visits within the previous year were assessed separately and not considered a healthcare exposure.

a Variables with a p-value < 0.1 were considered for the variable selection process in a multivariable regression analysis.

## 4 Discussion

In this study, the overall prevalence of CRE colonization was 8.1% and ESBL-E colonization was 73.6%. In our proxy group for community acquired MDR colonization (n = 20), no patient carried CRE, whereas 80% were colonized with ESBL-E. Hospitalization within the previous year was the sole factor significantly associated with ESBL-E colonization.

To our knowledge, rectal colonization by CRE has not been previously reported in Peru. In other countries, the reported prevalence of CRE colonization among ED patients has ranged from 0.08% to 6.8% [11,15].

An alarmingly high prevalence of rectal ESBL-E colonization was found. In Peru, rectal or fecal colonization by ESBL-E has been studied in outpatient pediatric populations, with reported prevalence between 58.7% and 70% [9,16] and in the adult outpatients in northern Peru, showing a prevalence of 85.8% [17]. In the proxy group for community acquired ESBL-E colonization (those with ≤48 h of admission and no prior healthcare exposure), the prevalence was markedly higher (80%) than the pooled global prevalence of 16.5% estimated in a recent meta-analysis [18], even surpassing the highest prevalence reported by countries in the South East Asia countries such as Vietnam (75.1%), Laos (70.2%) and China (58.5%).

Patients with a history of hospitalization within the previous year had 23% higher prevalence of ESBL-E colonization than those without this self-reported exposure. This finding aligns with results from previous studies in other countries [12,15,[17], [18], [19], [20], [21]] and supports reports on the persistence of colonization one year after patient follow-up [8]. Other previously reported risk factors for ESBL-E colonization such as hemodialysis use, presence of an intravascular catheter, recent antibiotic therapy, or use of corticosteroids [12,15,[17], [18], [19], [20], [21]] were not significantly associated to ESBL-E colonization in our study, although this might be due to the small sample size. In addition, we were underpowered to explore factors associated with CRE colonization due to the small number of CRE-colonized patients.

Our study has some limitations. First, enrollment was suspended prematurely in March 2020 due to the COVID-19 pandemic, before reaching the target sample size. However, the observed prevalence was higher than initially anticipated, and the precision of our estimates remained within an acceptable range. Nevertheless the reduced sample size, may have limited the statistical power to detect associations in the exploratory bivariate and multivariable analyses. Second, missing categorical data were handled via single imputation using the modal category (“No”) to maximize the number of observations included in the regression analysis. While this approach might have introduced misclassification bias if missing values were incorrectly designated as “No”, sensitivity analyses comparing the complete case dataset (130 cases) and imputed data analysis (148 cases) consistently identified "hospitalization within the previous year" as the sole predictor of ESBL-E colonization (Supplementary Table 3) suggesting that our primary findings are robust.

Third, the exclusion of 155 potential participants (37.5%) due to inability to provide informed consent may have introduced selection bias, since these excluded individuals were generally older, presented with altered mental status and had a more severe course of disease at the time of screening, meaning that their exclusion could have led to an underestimation of both ESBL-E and CRE colonization rates, as these patients often presents with multimorbidity, frequent exposure to healthcare facilities and invasive medical interventions (e.g., indwelling urinary catheters) that facilitate colonization by multidrug resistant pathogens. Fourth, individual-level demographic data was not collected for individuals who declined participation, further limiting our capacity to evaluate selection bias. Fifth, exposure history (e.g., prior hospitalization, antibiotic use) was self-reported, exposing the data to potential recall and interviewer biases. Additionally, since three quarters of participants were screened after a 48-h stay in the ED, it is challenging to definitively determine community acquisition or early nosocomial transmission within the ED. Nevertheless, the similar prevalence of ESBL-E colonization between patients screened before and after 48 h suggests possible limited early transmission in the ED. Conversely, the pattern of CRE colonization pattern is more concerning for possible early acquisition in the ED as all CRE cases detected within the first 48 h occurred in patients with clear prior healthcare exposure, whereas the majority of the cases detected after 48 h occurred in patients without prior healthcare exposure.

Finally, because this was a single-center study, the generalizability of these CRE and ESBL-E prevalence rates to other hospitals or regions in Lima or Peru may be limited. Multicenter studies are required to address these limitations and to monitor the prevalence estimates of ESBL-E and CRE colonization in the Peruvian population.

In conclusion, the extraordinarily high prevalence of ESBL-E colonization, including those without a prior history of health care exposure, underscores the current challenge for clinical management and antimicrobial stewardship programs in hospitals in Lima, Peru.

Additionally, the finding that 8% of patients admitted to the ED were colonized with CRE uncovers an overlooked reservoir for potential nosocomial outbreaks. These results highlight the urgent need to strengthen infection prevention and control strategies and to consider targeted active surveillance screening upon ED admission. However, our data also suggests an additional complexity since restricting screening solely to patients with a history of prior hospitalization would fail to identify a substantial proportion of carriers. Therefore, future epidemiological and molecular studies are needed to better characterize transmission dynamics and guide targeted, effective interventions.

## Ethical approval statement

This study was approved by the ethical committee of Hospital Cayetano Heredia (approval no. 80-2019, date August 08th, 2019) and Universidad Peruana Cayetano Heredia (approval no. 501-26-19, date October 24th, 2019).

## Funding

This study was funded by the Belgian Directorate for Development Cooperation (DGD) through the Framework Agreement 4 between the Belgian DGD and the 10.13039/100018370Institute of Tropical Medicine, Belgium.

## CRediT authorship contribution statement

**Gustavo Gutierrez Batallanos:** Conceptualization, Data curation, Formal analysis, Investigation, Methodology, Project administration, Validation, Writing – original draft, Writing – review & editing. **Alvaro Madrid:** Conceptualization, Data curation, Formal analysis, Investigation, Methodology, Project administration, Resources, Writing – original draft, Writing – review & editing. **Edwin Varillas:** Conceptualization, Data curation, Formal analysis, Investigation, Methodology, Project administration, Validation, Writing – original draft, Writing – review & editing. **Noemi Hinostroza:** Data curation, Formal analysis, Investigation, Methodology, Supervision, Validation, Writing – original draft, Writing – review & editing. **Fiorella Krapp:** Conceptualization, Data curation, Formal analysis, Funding acquisition, Investigation, Methodology, Project administration, Supervision, Validation, Writing – original draft, Writing – review & editing.

## Declaration of competing interest

The authors declare that they have no known competing financial interests or personal relationships that could have appeared to influence the work reported in this paper.
