# Supplementary material for: High prevalence of intestinal colonization by beta-lactam resistant Enterobacterales in patients admitted to an emergency department in Lima, Peru
Source: New Microbes New Infect. 2026 Aug 4;73:101824. doi: 10.1016/j.nmni.2026.101824 (PMC13503078; doi:10.1016/j.nmni.2026.101824)
Supplement: Supplementary file 1 [file mmc1.docx]

**Supplementary Table 1.** Prevalence of CRE  and ESBL-E colonization stratified by time since ED admission and prior healthcare exposure. in patients admitted to the emergency department of a tertiary care hospital in Lima, Peru

| **Time since admission (hours)** | **Prior healthcare exposure** | **Colonized by** | | | | **Colonized patients**  **(CRE, ESBL-E  or both)** | | **Non - colonized patients** | |
| --- | --- | --- | --- | --- | --- | --- | --- | --- | --- |
|  |  | **CRE** | | **ESBL-E** | |  |  |  |  |
|  |  |  |  |  |  |  |  |  |  |
|  |  | **(N=12)** | | **(N=109)** | | **(N=112)** | | **(N=36)** | |
|  |  | **n** | **(%)** | **n** | **(%)** | **n** | **(%)** | **n** | **(%)** |
| ≤ 48 | Yes (n=17) | 3 | (17.6) | 14 | (82.4) | 14 | (82.4) | 3 | (17.6) |
|  | No (n=20) | 0 | (0.0) | 16 | (80.0) | 16 | (80.0) | 4 | (20.0) |
| > 48 | Yes (n=45) | 2 | (4.4) | 34 | (75.6) | 35 | (77.8) | 10 | (22.2) |
|  | No (n=66) | 7 | (10.6) | 45 | (68.2) | 47 | (71.2) | 19 | (28.8) |
|  |  |  |  |  |  |  |  |  |  |

*CRE, Carbapenem-resistant Enterobacterales; ESBL-E, ESBL-producing Enterobacterales. There were 9 patients co-colonized with ESBL-E and CRE*
