# Supplementary material for: High prevalence of intestinal colonization by beta-lactam resistant Enterobacterales in patients admitted to an emergency department in Lima, Peru
Source: New Microbes New Infect. 2026 Aug 4;73:101824. doi: 10.1016/j.nmni.2026.101824 (PMC13503078; doi:10.1016/j.nmni.2026.101824)
Supplement: Supplementary file 2 [file mmc2.docx]

**Supplementary Table 2.** Characteristics of CRE colonized patients

| ***Case*** | ***Sex*** | ***Age*** | ***Time following admission*** | ***Diagnosis on admission*** | ***Comorbidities*** | ***Previous Antibiotic use*** | ***Previous healthcare exposure*** | ***ESBL-E isolates*** | ***CRE isolates*** |
| --- | --- | --- | --- | --- | --- | --- | --- | --- | --- |
| **1** | F | 67 | 26 h | UTI | Diabetes, ESRD on dialysis | Vancomycin, previous month | Dialysis + Hospitalization, previous month | *E. coli* | *K. pneumoniae* |
| **2** | F | 81 | 28 h | Respiratory failure | ESRD on dialysis | Cephalosporin, macrolides, penicillin, previous month | Hospitalization, previous month | *E. coli* | *E. coli* |
| **3** | M | 79 | 46 h | Respiratory failure | Lung disease | Cephalosporin, macrolides, clindamycin, previous month | Hospitalization within the previous year. | *E. coli* | *E. coli* |
| **4** | F | 79 | 55 h | Intra Abdominal sepsis | No | Unknown | Hospitalization, previous 02 months. | **-** | *E. coli.* |
| **5** | F | 62 | 4 d, 17 h | Stroke | Cervix cancer | No | No | **-** | *K. pneumoniae* |
| **6** | F | 49 | 6 d | Bronchiectasis | Diabetes | No | Hospitalization previous 5 months | *E. coli* | *K. pneumoniae*  *E. coli* |
| **7** | F | 84 | 6 d, 8 h | Septic shock | ESRD on dialysis | No | No | *E. coli*  *K. pneumoniae* | *E. coli*  *K. pneumoniae* |
| **8** | F | 69 | 9 d, 21 h | Massive pleural effusion | Asthma | Penicillin, previous month | Outpatient | *E. coli* | *K. pneumoniae (Strain 1 and 2)* |
| **9** | M | 95 | 11 d, 13 h | Stroke | No | No | Outpatient | *K. pneumoniae (Strain 1 and 2)* | *Enterobacter* |
| **10** | M | 84 | 11 d, 18 h | Stroke | Lung Cancer | No | No | *K. pneumoniae* | *E. coli*  *K. pneumoniae* |
| **11** | F | 55 | 12 d | SSTI | No | No | Outpatient | *E. coli* | *K. pneumoniae* |
| **12** | F | 65 | 12 d, 22 h | Stroke | No | No | No | **-** | *K. pneumoniae* |

*UTI, Urinary Tract Infection; ESRD End stage renal disease; SSTI skin and soft tissue infection, CRE, Carbapenem-resistant Enterobacterales; ESBL-E, ESBL-producing Enterobacterales*
