# Supplementary material for: High prevalence of intestinal colonization by beta-lactam resistant Enterobacterales in patients admitted to an emergency department in Lima, Peru
Source: New Microbes New Infect. 2026 Aug 4;73:101824. doi: 10.1016/j.nmni.2026.101824 (PMC13503078; doi:10.1016/j.nmni.2026.101824)
Supplement: Supplementary file 3 [file mmc3.docx]

**Supplementary Table 3.** Model comparison using the Likelihood Ratio (LR) test for predicting ESBL-E colonization among patients in the Emergency Department of a tertiary care hospital in Lima: Complete Case Analysis and Imputed Dataset.

|  | **Variable** | **LR test p-values** | | | |
| --- | --- | --- | --- | --- | --- |
|  |  | **Complete case analysis (n=130)** | | **Imputed dataset (n=148)** | |
|  |  | **Level 1** | **Level 2** | **Level 1** | **Level 2** |
| 1 | Age category (≤ 75 years, > 75 years) | 0.105 | 0.052 | 0.126 | 0.074 |
| 2 | Chronic liver disease | 0.080 | 0.100 | 0.055 | 0.067 |
| 3 | Chronic pulmonary disease | 0.156 | 0.223 | 0.119 | 0.165 |
| 4 | Hospitalization within the previous year | **0.023** | ─ | **0.036** | ─ |
| 5 | Previous outpatient visit | 0.056 | 0.054 | 0.060 | 0.069 |

*Variables with p < 0.1 in the bivariate analysis were entered into a manual forward selection process. Only ‘Hospitalization within the previous year’ met the selection criteria (p < 0.05) in both analyses and remained in the final model. As the analysis with the imputed dataset included a larger sample and provided more precise results, it was used to report the prevalence ratio*.
